# Using an internet-based platform to provide online and offline healthcare services for discharged patients

**DOI:** 10.1186/s12912-024-02161-y

**Published:** 2024-07-16

**Authors:** Lei Cui, Zirong Tong, Rong Wang, Xiaoping Fang, Longxiu Liu

**Affiliations:** 1 Pancreas Center, First Affiliated Hospital with Nanjing Medical University, Nanjing, China; 2 Nursing Department, First Affiliated Hospital with Nanjing Medical University, Nanjing, China

**Keywords:** Chronic diseases, Internet-based healthcare, Continuing care, Home care

## Abstract

**Background:**

Continuing care is needed for the growing number of chronically ill patients who struggle with health problems after discharge. This study aims to elucidate the development process, functionalities, service protocols, and utilization of an Internet Plus Care (IPC) platform devised by our hospital to offer healthcare services to discharged patients.

**Methods:**

This was a mixed study. After describing the development process, function and usage of IPC platform, we retrospectively collect data such as the general information of service recipients and service providers, service items, and service prices through the IPC platform from January 2021 to September 2023 to characterize these services.

**Results:**

69 nurses delivered a total of 788 services to 211 patients through the IPC platform. The majority of services (*N* = 652, 82.7%) were delivered offline, with almost half of the recipients (*N* = 384, 48.7%) being elderly individuals. 46.4% of nurses provided services ≥ 3 times. Furthermore, 26.5% of patients received services three or more times. Notably, patients’ care requirements exhibited variations across age groups, with wound care (*n* = 243, 63.3%), pressure injuries care (*n* = 50, 13.0%), and replacement of indwelling nasogastric tubes (*n* = 20, 5.2%) emerging as the top three services favored by the elderly.

**Conclusions:**

The IPC platform demonstrates potential in delivering diverse health services to patients; however, the involvement of nurses and patients needs to be enhanced. It is necessary to implement relevant safeguard policies to promote the effective use of IPC platform for health management of discharged patients in the future.

**What does this paper contribute to the wider global clinical community?:**

- The prevalence of chronic diseases is on the rise, and patients with chronic diseases continue to struggle with health problems after discharge and require extended treatment and rehabilitation. Our study proves that IPC platform presents a promising avenue for addressing these challenges.
- It is anticipated that governmental authorities will undertake comprehensive policy, legislative, and medical insurance reforms to facilitate the extensive adoption of IPC platform-based services.

**Supplementary Information:**

The online version contains supplementary material available at 10.1186/s12912-024-02161-y.

## Introduction

Chronic diseases are defined as conditions lasting for one year or more, necessitating continuous medical attention, limiting activities of daily living, or both [1]. Due to the accelerated aging of populations, increasing prevalence of behavioral risk factors, and extended survival of patients, there is a growing population afflicted by chronic diseases. According to the National Health Commission, over 180 million elderly individuals in China suffer from chronic diseases, with the percentage of those affected by one or more chronic diseases soaring to as high as 75% [2]. Chronic diseases account for a staggering 88.5% of all fatalities in China [3]. This imposes substantial healthcare and economic burdens on both society and families.

The management and rehabilitation of chronic disease patients require a protracted process, with lingering health issues post-discharge. In a prospective cohort study examining post-discharge issues and requirements, 91.8% of patients reported challenges related to at least one physical ailment, while 73.8% sought additional information regarding one or more facets of their care [4]. Another study indicates that 83.5% of older adult patients are aware of, and 55.7% request, at least one form of post-discharge healthcare service [5]. Research has established that extended care services for post-discharged patients can significantly enhance prognosis, encompassing improved quality of life, reduced readmission rates, and extended survival durations [6–8]. While patients receive professional care during hospitalization, the question arises: Who will attend to their needs after discharge? Despite the convenience and cost-effectiveness of treating numerous conditions by primary care providers, many patients exhibit reluctance due to concerns about the expertise of healthcare professionals and the quality of care provided [9]. Patients often seek care at tertiary hospitals, even for minor symptoms [9].

In the era of rapid information technology advancement, numerous high-level hospitals have leveraged mobile health platforms to offer post-discharge patients health consultations, information recording, online guidance, and various remote preventative, therapeutic, and rehabilitative health services [8, 10, 11]. The widespread adoption of these online services as supplementary medical resources has significantly benefited patients, partly fulfilling their health needs. However, what about health services that cannot be delivered online?

To overcome the limitations of online services, services based on the Internet Plus Care (IPC) platform present a promising solution. It primarily entails medical institutions utilizing in-house registered nurses to provide care services for discharged patients or individuals with specific health conditions and mobility impairments, through a “combined online and offline services” approach facilitated by the internet and other information technologies [12]. While the nomenclature of IPC services may vary among different countries, it has been developed and widely implemented in numerous nations [13, 14]. In China, IPC services were introduced relatively late, with official initiation occurring in 2019 following the issuance of the National Health Commission’s Notice on Launching IPC Service Pilot Programs and the IPC Service Pilot Program Plan [15, 16]. These policies designated six provinces and municipalities as IPC service pilot locations, and subsequent national policies have underscored the priority of extending continuous care services and expanding IPC services [15, 16].

In recent years, driven by national policies, several prominent hospitals have actively explored transitional care models established on IPC platforms. As a comprehensive tertiary hospital in Jiangsu Province and one of the early IPC service pilot regions, our hospital formally adopted the IPC platform in 2021. This study aimed to initially outline the developmental process, functionalities, and utilization patterns of the IPC platform. Additionally, it sought to characterize both online and offline services by retrospectively gathering data, including general patient information, details of service items, service fees, and general nurse profiles involved in providing services through the IPC platform from January 2021 to September 2023. The significance of this study is that on the one hand, it can provide methodological guidance for other hospitals to achieve online and offline medical cooperation through Internet technology, so as to provide more comprehensive medical services for patients. On the other hand, the defects of IPC-based platform services implemented in our hospital can be found, which can provide a basis for better service improvement in the future.

## Methods

### Study design

To achieve our research objectives, we used a mixed research approach [17]. During the IPC platform design phase, we organized a focus group with all stakeholders including patients, engineers, nurses, etc., to discuss the needs of home service and IPC platform design. After the initial design of the IPC was completed, patients and nurses were recruited for functional testing, and individual interviews were conducted to collect their feelings and opinions on the use of the IPC platform for continuous improvement. In the IPC platform application phase, we used a retrospective study to investigate the use of the IPC platform.

### Development process, functionalities, and description of the IPC platform

The IPC platform was meticulously developed on the basis of medical informatics theory, while incorporating the principles of holistic nursing and the delivery of high-quality healthcare services. As for the developmental journey of the IPC platform, the process commenced with the establishment of a dedicated working committee. This committee was comprised of key figures within the hospital’s administrative hierarchy, including the Vice President overseeing nursing affairs, the Director of the Nursing Department, the Deputy Director of the Nursing Department in charge of IPC services, as well as several leaders of specialized care units, such as cancer care, rehabilitation training, and skin care. The working committee collectively resolved to collaborate with the engineering team responsible for the platform’s development. Finally, the completed platform included three main interfaces: patients, nurses, and administrator. The main functions of the patient interface are user registration, appointment service, payment and evaluation service. The main functions of the nurse interface were service response, patient assessment, nursing record, start positioning, and medical waste disposal record. The main functions of the administrator interface were patient and nurse information review, service allocation, nurse positioning, and service information record.

Items of service within the IPC platform were meticulously curated in alignment with criteria emphasizing substantial demand, safety, efficacy, minimal operational risks, ease of implementation, compliance with disinfection and isolation standards, and low incidence of adverse reactions. Services encompassing high-risk procedures such as intravenous infusions, administration of oral or injectable narcotic medications, specialized management involving psychotropic substances, radioactive materials, medically toxic agents, and invasive medical procedures, were stringently prohibited. Finally, the platform can provide patients with basic care, rehabilitation care, specialized care, obstetric care, neonatal care and online consultation, as detailed in Additional Fig. 1.

In order to ensure patient safety and uphold the quality of care, detailed selection criteria were devised for prospective service providers. Registered nurses meeting the following criteria could apply to become “appointed nurses” for IPC services: (1) A minimum of five years of clinical practice experience, (2) Holding a nurse or higher professional technical title, (3) Possessing outstanding professional attributes including a strong sense of responsibility, compassion, self-regulation, and a robust commitment to safety and adaptability, (4) Exhibiting organizational, coordination, and communication competencies, and (5) Attaining specialist nurse qualifications at or above the municipal level for specific service domains, such as diabetes and chronic heart failure management. Approved candidates underwent public courses and specialized technical training, subject to endorsement by the Head Nurse, the leader of the respective specialized care group, and the Director of the Nursing Department. Subsequently, successful completion of all training, including theoretical and skills assessments, led to the acquisition of IPC service qualification certificate. To maintain the quality of care, these qualified nurses are reassessed annually with theoretical and practical examination results and satisfaction ratings from service recipients. The examination was organized by the nursing department, and the evaluation results of service recipients could be extracted on the IPC platform. Nurses were disqualified if they failed the examination or had two unsatisfactory evaluations during their service. The admission and withdrawal procedures for service providers are elucidated in Additional Fig. 2.

Access to IPC services, due to finite nursing human resources, is currently extended exclusively to patients who have previously visited our hospital. Additionally, for the safety of service personnel, patients must not exhibit infectious diseases or possess aggressive behavioral traits associated with mental disorders. After the patients signed the informed consent form, the nurses provided the printed IPC platform operation manual to the discharged patients, and carefully informed the patients about the use of the IPC platform to ensure that the patients would use the IPC platform. The operation manual also includes a service hotline that patients can call after discharge if they have any questions. Subsequently, patients, guided by the operational manual, enter personal information and complete registration on the IPC platform, thereby preparing for the subsequent scheduling of IPC services.

**Fig. 1:**
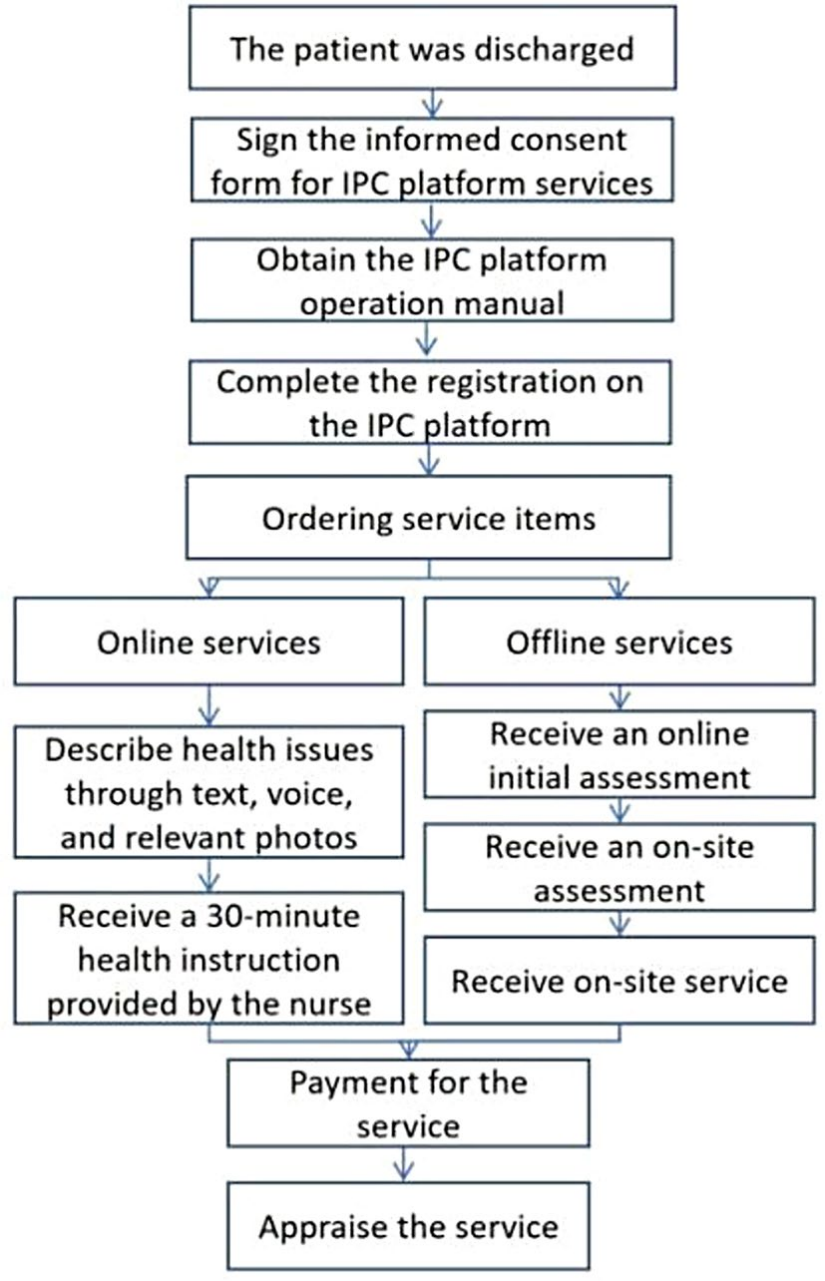
The service workflow based on Internet Plus Care platform

The service processes for online and offline modes exhibit notable distinctions (see Fig. 1). Commencing the procedure is the initial registration on the IPC platform. Users are required to provide fundamental personal information in accordance with the platform’s registration interface requirements, culminating in the successful registration process. Subsequently, patients are empowered to place orders for service items via the IPC platform. In the event that a patient opts for online services, they are afforded the capability to articulate their health concerns utilizing text, voice, and pertinent visual documentation. The nurse assigned to the order dedicates 30 min to provide requisite guidance. Alternatively, for patients selecting offline services, they are prompted to furnish details such as the service address and a comprehensive description of their medical history, comprising outpatient or inpatient treatment records from our hospital. This initiates a preliminary evaluation of the patient’s eligibility for service. The managerial team undertakes a review of the patient’s personal particulars and appointment details, culminating in an initial assessment of the patient’s suitability for on-site service. If this evaluation yields a positive outcome and the patient has designated specific service personnel, the order is directly dispatched to the corresponding nurses. Conversely, if no personnel preferences have been specified, a process for order allocation is initiated. The ensuing stage involves the execution of on-site assessment and service delivery. Upon receipt of the order, the nurse meticulously assembles the requisite medical supplies, aligned with the patient’s service requirements. The nurse then selects the appropriate mode of transportation contingent on the patient’s residential proximity. During the on-site visit, nurses conduct a thorough evaluation to ascertain the patient’s fitness for on-site service. In cases where on-site service is deemed unsuitable, the service is curtailed, with the corresponding evaluation fee duly received. Conversely, if the patient is deemed suitable, the designated service provisions are carried out. Upon the conclusion of both online and offline service modalities, patients are provided the opportunity to appraise the quality of service received.

To guarantee the safety of both nurses and patients, the IPC platform incorporates an array of protective functions. These functions are elaborated upon as follow: (1) User Authentication: Users are mandated to register using verifiable personal information, including submission of medical records and copies of both sides of their identity card for administrator review. Additionally, the platform employs facial recognition during care service bookings, automatically comparing the user’s live image with the previously provided identity photo to ensure congruence. (2) Real-time Positioning: The global positioning system is utilized to continuously monitor the real-time location of nurses, providing precise location information. (3) One-Touch Emergency Alert: In situations where nurses perceive danger or feel threatened, they can immediately trigger an emergency alert. This alert interfaces with the public security system and automatically reports the incident to law enforcement. (4) Real-time Video Recording: Nurses are required to record video footage from the moment they enter a service recipient’s home until the conclusion of the service. Platform administrators can access and monitor these videos in real-time. In instances of any anomalies, administrators can take appropriate actions, including contacting law enforcement. We have also taken some measures against common social engineering attacks on IPC platforms. These include backing up data regularly to prevent the loss of important data, conducting regular security checks to detect vulnerabilities and viruses, setting complex passwords, and updating them regularly to prevent them from being leaked.

In terms of fee structures, services rendered through the IPC platform operate under two modes: online and offline on-site services. Online services incur a flat fee of 2.8 dollars per session. Meanwhile, charges for offline on-site services encompass expenses of care, transportation, and consumables. The fees for care services were formulated in alignment with the pricing standards for medical services in Jiangsu province, approved nurse remuneration rates, the level of risk associated with nursing services, and were subsequently recorded with the price examination and approval department. Transportation fees were calculated based on urban taxi fare per kilometer, while consumables were procured by the hospital and personally delivered to patients’ homes by nursing staff during service delivery. All fees were uniformly collected by the platform, and nurses were strictly prohibited from imposing additional charges on patients without authorization.

### Data collection

The demographic and professional information of nurses possessing service qualifications was extracted from the nursing information system. This dataset encompassed variables such as age, gender, educational attainment, professional titles, nursing level, departmental affiliation, and the number of service credentials held. Concurrently, service-related data spanning from January 2021 to September 2023 was obtained from the IPC platform administrator, comprising details such as the age of service recipients, service duration, service category, associated costs, and the identities of service providers.

### Data analysis

The data analysis was conducted by the principal investigator, Cui L, who underwent specialized training for this purpose. Additionally, a statistics PhD from Nanjing Medical University was consulted to ensure the accuracy of the statistical methodologies employed. Descriptive statistics were used to analyze the data. Categorical variables are described as frequencies and percentages, and numerical variables are described as means and standard deviations or interquartile ranges, depending on whether they are normally distributed. All data were analyzed using the Statistical Package for the Social Sciences (SPSS), Version 26.0.

## Results

### Basic characteristics of nurses with service qualifications

A total of 209 nurses were qualified to provide 50 services through the IPC platform. All of them were female with a median age of 39 years. The majority held a bachelor’s degree or higher and possessed an intermediate or advanced professional title. A total of 69 nurses had provided services based on IPC platform for patients. There was no significant difference in age, gender, years of working, education level, technical title, nurse level and department between nurses who had provided services and those who had not. Detailed characteristics of these nurses are presented in Additional Table 1.

## Number of IPC services

**Fig. 2:**
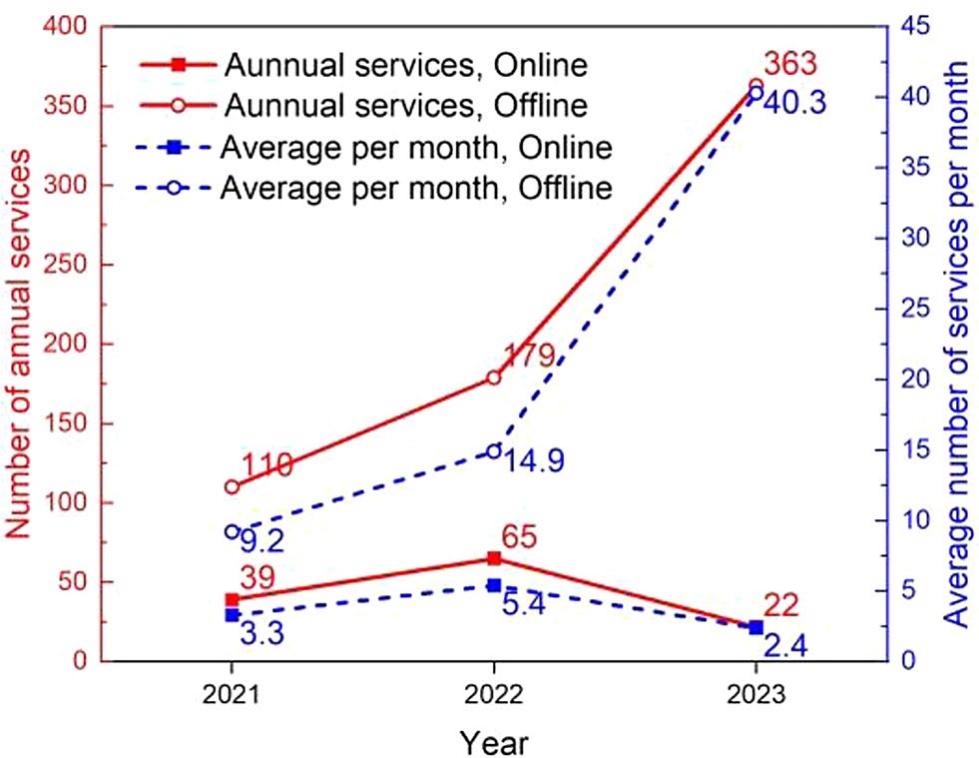
Number of services based on the Internet Plus Care platform

Between January 2021 and September 2023, a total of 788 services were executed utilizing the IPC platform, with 635 of them being on-site offline services. Figures summarizing the total number and monthly average per year for these services are presented in Fig. 2. The utilization of on-site offline services via the IPC platform displayed a year-on-year increase. The average monthly offline services in 2023 were 4.4 times and 2.7 times higher than in 2021 and 2022, respectively. Conversely, online services via the IPC platform peaked in 2022 and then receded below the 2021 level. The average monthly online service instances in 2021, 2022, and 2023 were 3.3, 5.4, and 2.4, respectively.

### Number of nurses providing services and patients accessing services

A total of 69 nurses delivered 788 online and offline services to 211 patients through the IPC platform. The highest number of services provided by a single nurse was 305 times, encompassing 260 instances of wound care, 40 cases of pressure injury care, four occasions of nasogastric tube replacement, and one occurrence of standard stoma care. Notably, nearly half of the nurses (*n* = 32, 46.4%) delivered services through the IPC platform on three or more occasions. Among the patients, the maximum number of services received by a single individual was 43, all of which were wound care. Specifically, 56 patients (26.5%) availed services on three or more occasions, while 36 patients (17.1%) utilized services five or more times. The details regarding the number of nurses providing services and patients accessing services are displayed in Additional Table 2.

### Distribution of services based on the IPC platform

**Fig. 3:**
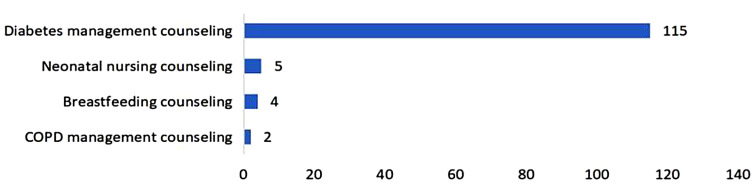
Number of online services based on the Internet Plus Care platform (*N* = 126)

**Fig. 4:**
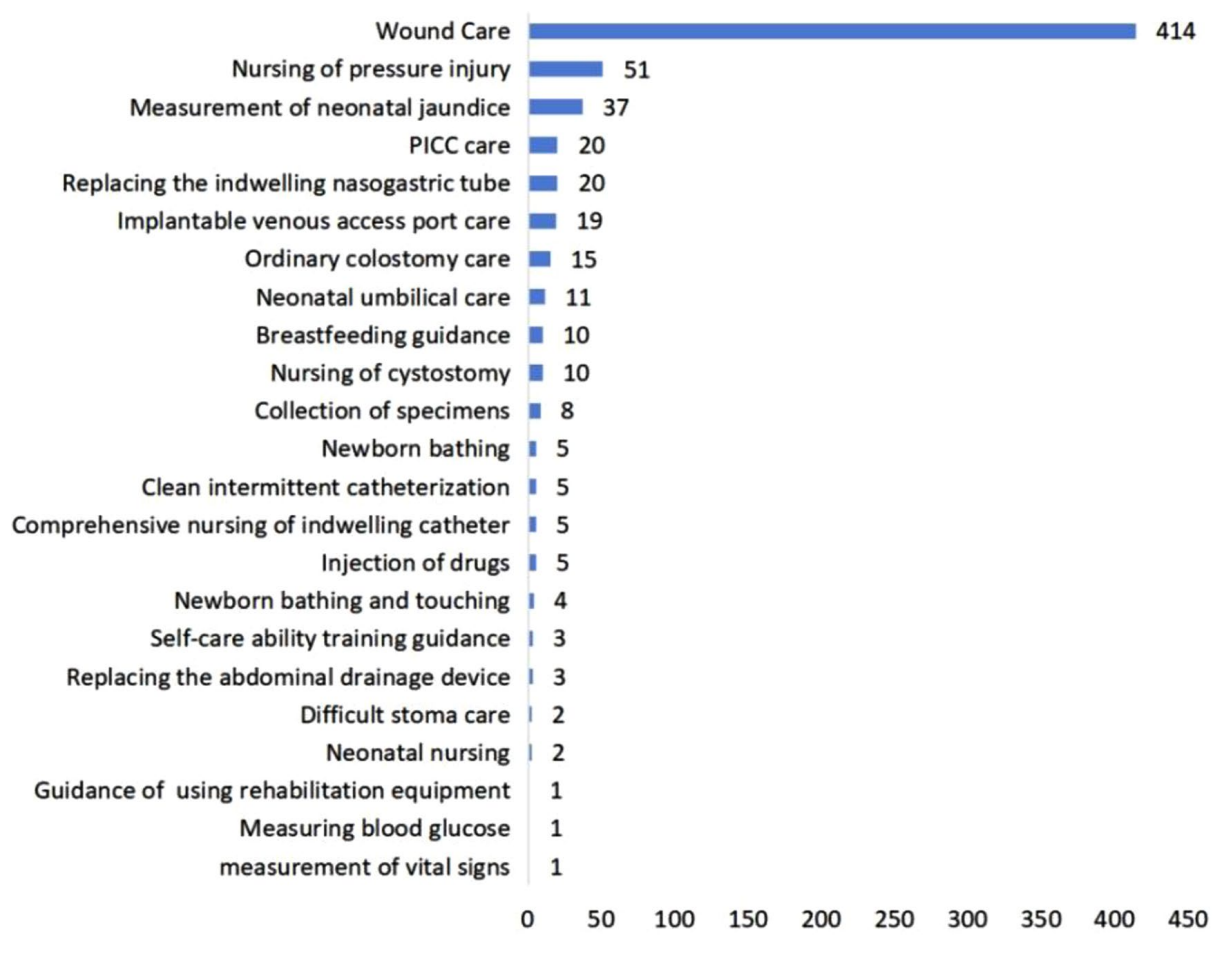
Number of offline services based on the Internet Plus Care platform (*N* = 652)

Figure 3 illustrates that out of 126 online services delivered through the IPC platform, diabetes management counseling (*n* = 115, 91.3%) constituted the majority. A total of 652 offline services were facilitated through the IPC platform. The top three were wound care (*n* = 414, 63.5%), nursing for pressure injuries (*n* = 51, 7.8%), and neonatal jaundice measurement (*n* = 37, 5.7%) (see Fig. 4).

We additionally elucidated the services availed by patients across different age groups via the IPC platform (see Additional Fig. 3 to 7). Among the 788 online and offline services, 57 were administered to infants and young children (up to 6 years old), 44 to teenagers (7 to 17 years old), 133 to young adults (18 to 40 years old), 165 to middle-aged individuals (41 to 65 years old), and 384 to the elderly (over 65 years old). For infants and young children (see Additional Fig. 3), services encompassed neonatal jaundice measurement (*n* = 37, 64.9%), neonatal umbilical care (*n* = 11, 19.3%), neonatal bathing (*n* = 5, 8.8%), and neonatal bathing and handling (*n* = 4, 7.0%). Among teenagers (see Additional Fig. 4), the predominant service was wound care (*n* = 39, 88.6%). For young adults (see Additional Fig. 5), the top three services were diabetes management counseling (*n* = 63, 47.4%), wound care (*n* = 46, 34.6%), and breastfeeding guidance (*n* = 9, 6.8%). Middle-aged individuals (see Additional Fig. 6) primarily received wound care (*n* = 86, 52.1%), diabetes management counseling (*n* = 41, 24.8%), and PICC care (*n* = 14, 8.5%). As indicated in Additional Fig. 7, the elderly predominantly sought wound care (*n* = 243, 63.3%), nursing for pressure injuries (*n* = 50, 13.0%), and indwelling nasogastric tube replacement (*n* = 20, 5.2%).

### Payment amount of online prescriptions

The cost of online consultation services via the IPC platform was 2.8 dollars per session. For offline on-site services, the expenses encompassed fees of transportation, consumables, and the service itself. The average costs were 6.7, 2.0, and 37.3 dollars, respectively, as presented in Table 1.

**Table 1:** Payment amount of services based on the Internet Plus Care platform

Items		Cost ($)
Online services	Service charge	2.8 ± 0
Offline services	Service charge	37.3 ± 5.0
	Cost of transportation	6.7 ± 5.1
	Cost of consumables	2.0 ± 6.1
	Total cost	46.0 ± 8.9

## Discussion

The UK and other developed countries have achieved a relatively advanced stage of development in “Internet nursing service”, “home-visiting nurses”, and transitional care [18–20]. Extensive efforts have been directed towards e-health and the digitization of home care, revealing significant potential for e-health services in this domain [18–20]. In contrast, the development of Internet nursing services in China has been relatively nascent. Due to different socioeconomic factors and cultural backgrounds, the service model based on IPC platform in our country is different from other countries. In contrast to home care models in many other countries that focus on providing continuous care to specific patients, China’s Internet-based platform often connects nurses with new patients for each service session, resulting in the establishment of new nurse-patient relationships [21, 22]. Furthermore, while home care in other countries is primarily provided by specialized agencies with full-time nurses, in China, nurses are affiliated with hospitals and deliver home care during their non-duty hours, rendering them part-time caregivers [23–25].

Our study indicates that since the inception of the IPC platform, a total of 788 service orders have been fulfilled, with the number of orders consistently rising. Notably, online consultation orders peaked in 2022 and then declined, while offline services surged in 2023, surpassing the combined total of the previous two years. This shift may be attributed to the Chinese government’s evolving COVID-19 containment policies. Initially, measures aimed at minimizing human contact and promoting online medical consultations were implemented to curb virus spread. However, as of December 2022, the Chinese government began to relax its COVID-19 control measures [26]. Simultaneously, increasing awareness and use of the IPC platform may have contributed to this shift.

In our investigation, a total of 209 nurses possessed qualifications for the IPC platform service, yet only 69 (33.0%) engaged in providing either online or offline services. Notably, nearly half of the nurses who did offer services did so on three or more occasions, with one nurse providing services up to 305 times. These findings suggest that while a majority of nurses have yet to participate in Internet services, a substantial proportion of those who have are inclined to continue doing so. This inclination could be attributed to perceived benefits associated with Internet nursing, such as increased income, affirmation and enhancement of nursing skills, and an elevated professional identity, sense of personal worth, and social standing among nurses [27]. It is noteworthy that akin to our findings, a prior study by He et al. also reported a similar trend, with approximately 30% of nurses expressing willingness to engage as online appointment nurses [28]. Although this study did not find that age, gender, education level, technical title, nursing level, and work department affected nurses’ implementation of services based on IPC platform, other previous studies have pointed out several factors may contribute to nurses’ reluctance [29–33]. Concerns over safety, including navigating unfamiliar locations, potential hostility from unfamiliar patients and their families, and increased occupational risks, are significant deterrents [29, 30]. Additionally, conflicts between work, family, and social roles play a role, exacerbated by a shortage of nurses in China and the resulting heavy clinical workloads [31, 32]. Finally, existing laws and regulations are insufficient, incentives are lacking, and issues such as effort-reward imbalances and inadequate promotion systems further erode nurses’ motivation [29, 33]. In the future, enhancing nurses’ participation enthusiasm is imperative, and this can be achieved through various approaches as discussed above.

Among the 211 patients utilizing services via the IPC platform, 56 patients (26.5%) availed services on more than three occasions, while 36 patients (17.1%) accessed services over five times. These statistics highlight a notable willingness among patients to engage with and recurrently utilize services offered through the IPC platform. Such eagerness could be attributed to the alleviation of inconvenience and challenges associated with in-person hospital visits [34]. However, it is crucial to acknowledge that previous studies have indicated relatively lower levels of participation in home-based services compared to hospital-based ones [35]. There are several barriers that prevent patients from participating in Internet-based care. Firstly, several patients have insufficient cognition and deviation of service model based on the IPC platform [36]. Their subjective perception that Internet-based care is lack of formalization, mainly for seriously ill patients, coupled with the fact that low education level and the elderly population is unlikely to use the Internet as a communication tool for health-related issues, which leads to low acceptance of Internet-based care by the general public [37, 38]. Secondly, the high cost and the unclear charging standards of internet-based services are important factors hindering their promotion [36]. Since transportation costs and time costs are included in addition to service fees, Internet-based care services cost many times more than regular hospital visits. At present, Internet-based care services are not covered by medical insurance in China, and are completely paid by patients out of their own pocket, resulting in contradictions between residents’ demand for services and their ability to pay. Furthermore, there is no uniform standard for service charges, and most of them are determined by the Internet care platform according to the service items, service time and service distance, which also makes many patients worry that the charges are unreasonable [39]. Learning from other countries, including Japan and Germany [14, 15], which integrate home care into separate long-term care insurance systems, or adopting the integrated care model of the United States where health insurance covers home care on a per capita basis [40], could provide insights into addressing this challenge. The inability to meet the diverse service needs of patients is also the reason that limits the promotion of Internet-based care services [36]. Due to the lack of medical equipment and specialist nurses, some items such as debridement and sputum suction, which are highly dependent on medical equipment and professional level, are limited. Not only that, the time of services based on the IPC platform is generally limited to the daytime, which cannot timely solve the sudden health problems of patients at night. Finally, patients were concerned about their safety. They believed that the quality of care of nursing staff was difficult to ensure, the home environment was difficult to meet the same requirements of the hospital environment, and there was a lack of timely support from the medical team when dealing with emergencies [36]. In the future, it is imperative for both the government and hospitals to collaborate towards fostering greater patient participation in Internet nursing service platforms.

The results of this study indicate that the care demands of patients vary across age groups, and older adults are the main population who make appointments for services based on the IPC platform. This finding closely aligns with the outcomes of prior research, which revealed that the “Internet plus Nursing” service platform predominantly caters to elderly patients, a demographic characterized by a significant prevalence of compromised self-care abilities [41]. This may be related to the aging population and more comorbidities in the elderly: According to the National Bureau of Statistics, by the end of 2022, the elderly population aged 60 or above in China numbered 280.04 million, accounting for 19.8% of the total population. In addition, 150 million elderly people suffer from chronic diseases, accounting for 65% of the total elderly population, and about 40 million elderly people are disabled or semi-disabled, resulting in a rapidly growing demand for home care [17, 42].

### Limitations

This study has several limitations. Firstly, during the IPC platform design phase, we only considered patients treated at our hospital as potential service users. In the future, we aim to expand our services to all patients needing home care. Secondly, due to time and regional constraints, the IPC platform was only applied in our hospital. We hope to promote and apply it widely across the province to verify its effectiveness, continually supplementing and improving the nursing service list based on user needs to enhance service quality.

### Future prospects

The IPC platform-based service model presents opportunities to enhance patient convenience and advance nurses’ careers. However, several areas require attention to optimize this care model. Firstly, the legal framework for Internet-based care services needs enhancement, including clear regulations on nurses’ working hours and benefits, defined professional roles and responsibilities, and provisions for addressing errors and accidents during service delivery. Secondly, the establishment of a tiered medical system, encompassing tertiary, secondary, and community hospitals, could facilitate training for community hospital nursing staff by higher-level hospitals, allowing community nurses to provide home care services, thereby broadening access to Internet-based care. Lastly, the inclusion of Internet-based care services in medical insurance coverage would alleviate patients’ financial burdens and promote the adoption of these services.

## Conclusions

In light of the progressively intertwined relationship between information technology and the healthcare sector, the Internet-based care model emerges as the forefront of future service model reform. Our study findings suggest that the IPC platform has the capability to deliver a multitude of services to diverse patient populations. However, there is a notable need for enhanced engagement of nurses and patients. Moving forward, it is anticipated that governmental authorities will undertake comprehensive policy, legislative, and medical insurance reforms to facilitate the extensive adoption of IPC platform-based services.

### Electronic supplementary material

Below is the link to the electronic supplementary material.

Supplementary Material 1

## Data Availability

The data of this study are available from the corresponding authors on reasonable request.
