# Supplementary material for: Using an internet-based platform to provide online and offline healthcare services for discharged patients
Source: BMC Nurs. 2024 Jul 16;23:486. doi: 10.1186/s12912-024-02161-y (PMC11251320; doi:10.1186/s12912-024-02161-y)
Supplement: Supplementary file 1 — Supplementary Material 1 [file 12912_2024_2161_MOESM1_ESM.docx]

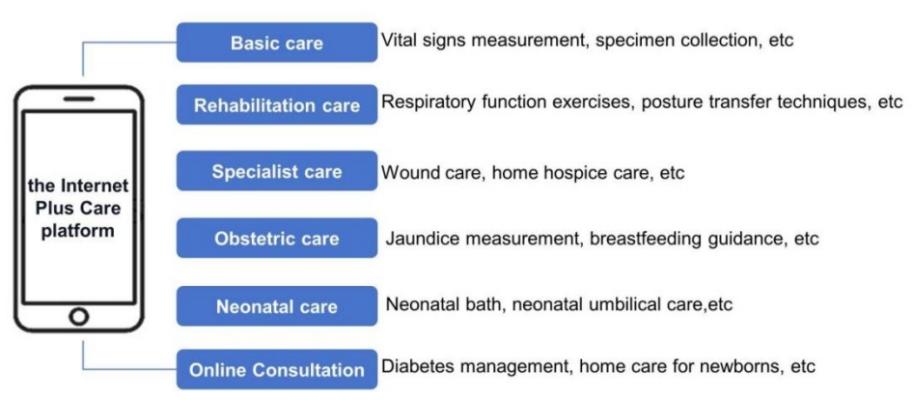


**Additional Fig. 1 Items of services provided by the Internet Plus Care platform**


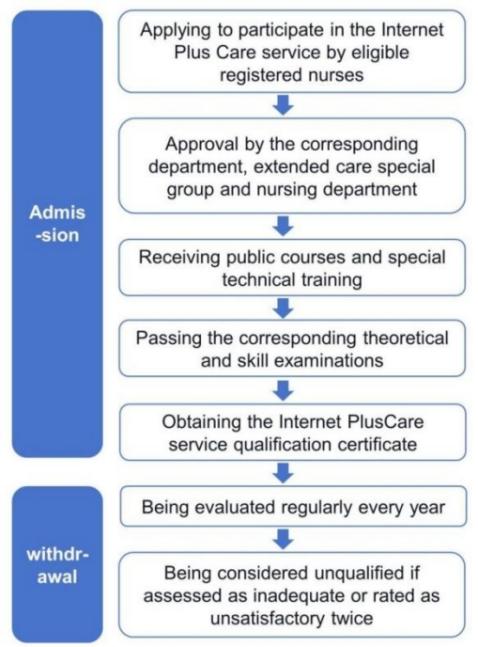


**Additional Fig. 2 The admission and withdrawal pathways of service providers**


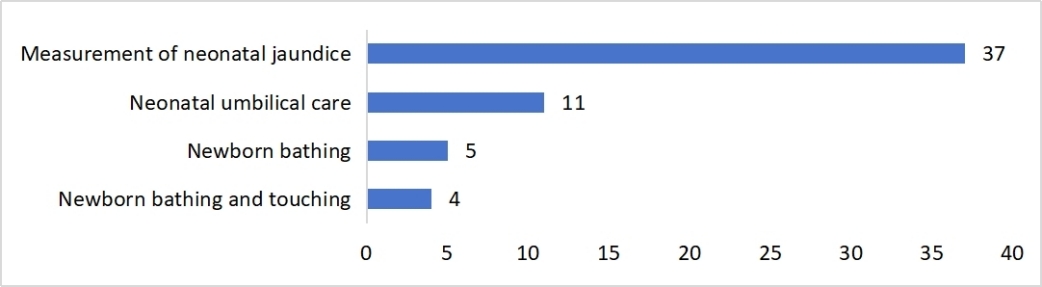


**Additional Fig. 3 Services for infants and young children through the Internet Plus Care platform (N=57)**


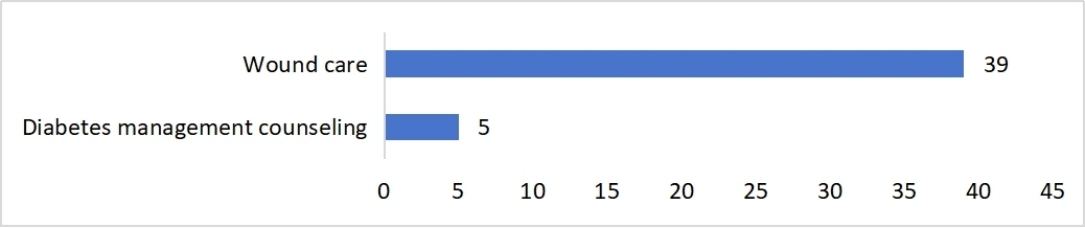


**Additional Fig. 4 Services for teenagers through the Internet Plus Care platform (N=44)**


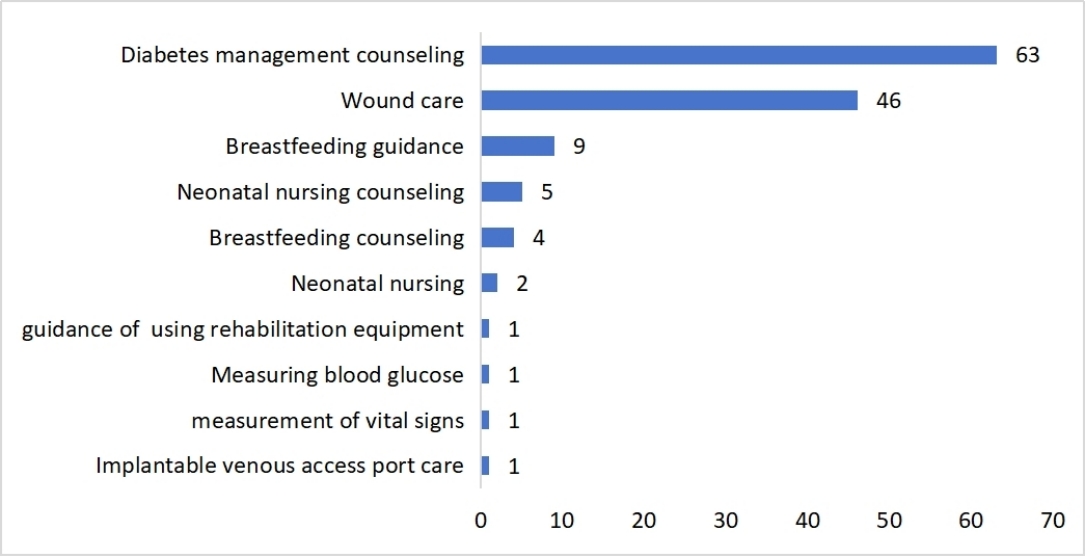


**Additional Fig. 5 Services for young adults through the Internet Plus Care platform (N=133)**


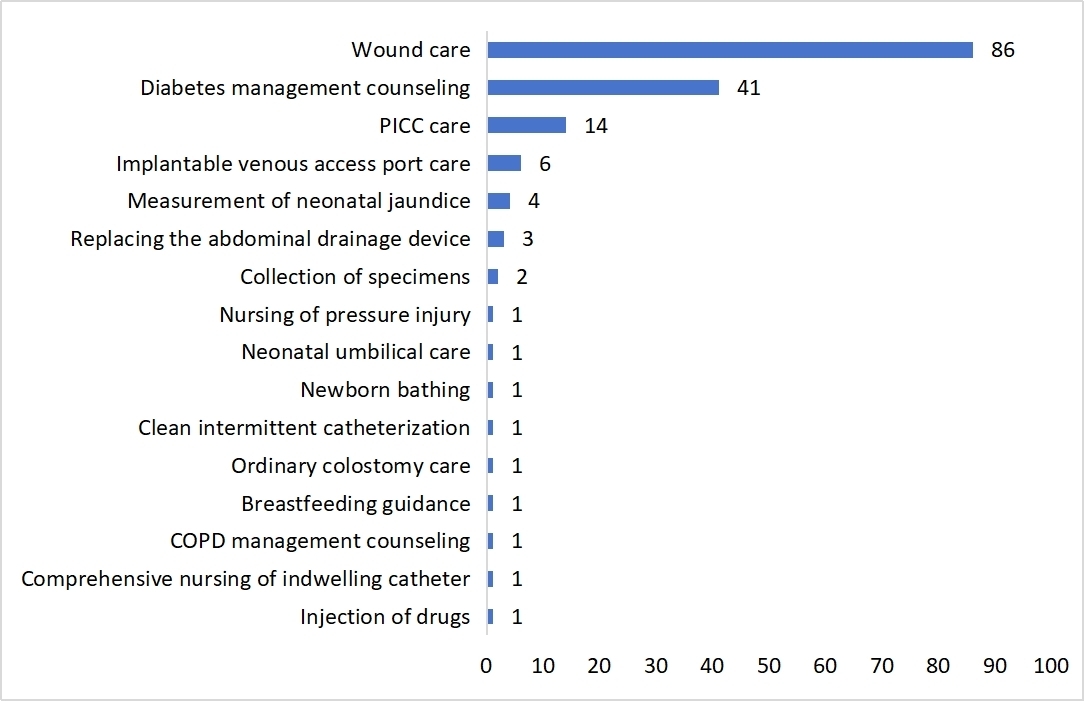


**Additional Fig. 6 Services for middle-aged people through the Internet Plus Care platform (N=165)**


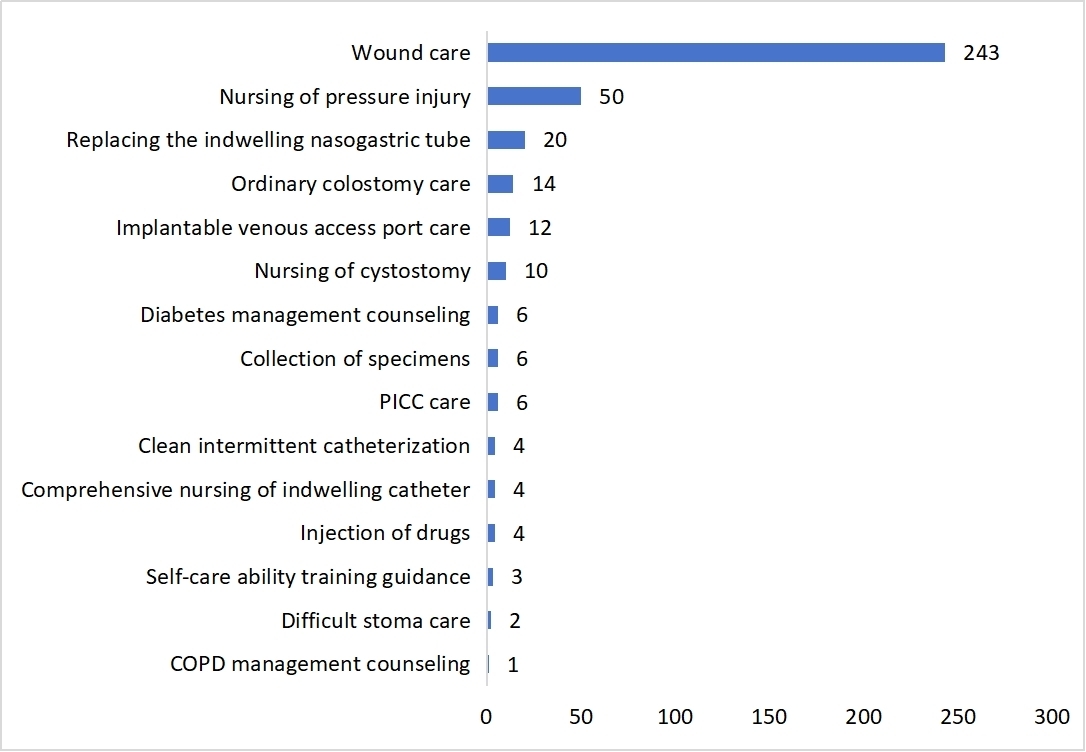


**Additional Fig. 7 Services for the elderly through the Internet Plus Care platform (N=384)**

**Additional table 1. Basic characteristics of nurses with service qualifications**

| **Characteristic** | | **Total**  **(n=209)** | **Provided services group(n=69)** | **Not provided services group (n=140)** | ***P*** |
| --- | --- | --- | --- | --- | --- |
| Female, n(%) | | 209(100.0) | 69(100.0) | 140(100.0) | -- |
| Age(years), IQR | | 39.0(34.0,42.0) | 39.0(34.0,42.0) | 38.0(33.0,42.0) | 0.608 |
| Years of working, IQR | | 15.0(9.0,19.0) | 15.0(9.5,19.0) | 14.5(9.0,19.8) | 0.816 |
| Educational level, n(%) | <bachelor degree | 4(1.9) | 2(2.9) | 2(1.4) | 0.600 |
|  | ≥Bachelor degree | 205(98.1) | 67(97.1) | 138(98.6) |  |
| Technical titles, n(%) | Junior | 71(34.0) | 22(31.9) | 49(35.0) | 0.610 |
|  | Medium-grade | 79(37.8) | 32(46.4) | 55(39.3) |  |
|  | Senior | 59(28.2) | 15(21.7) | 36(25.7) |  |
| Nursing level, n(%) | N1 | 2(1.0) | 0(0.0) | 2(1.4) | 0.197 |
|  | N2 | 90(43.0) | 25(36.2) | 65(46.4) |  |
|  | N3 | 83(39.7) | 34(49.3) | 49(35.0) |  |
|  | N4 | 34(16.3) | 10(14.5) | 24(17.1) |  |
| Department, n(%) | Internal medicine | 31(14.8) | 9(13.0) | 22(15.7) | 0.645 |
|  | Surgery | 60(28.7) | 18(26.1) | 42(30.0) |  |
|  | Geriatrics | 37(17.7) | 11(15.9) | 26(18.6) |  |
|  | Others | 81(38.8) | 31(44.9) | 50(35.7) |  |
| Number of service qualifications, IQR | | 2(1,5) | 3(1,5) | 2(1,5) | 0.052 |

**Abbreviations:** IQR, Inter-quartile Range

**Additional table 2. Number of nurses providing services and patients accessing services**

| **Number of services** | **Number of nurses providing services** | **Number of patients accessing services** |
| --- | --- | --- |
| <3 | 37 | 155 |
| 3~4 | 11 | 20 |
| 5~9 | 10 | 24 |
| 10~19 | 7 | 8 |
| 20~29 | 1 | 2 |
| 30~39 | 0 | 1 |
| 40~49 | 2 | 1 |
| ≥50 | 1 | 0 |
| Total | 69 | 211 |
